# Health care restructuring and family physician care for those who died of cancer

**DOI:** 10.1186/1471-2296-6-1

**Published:** 2005-01-04

**Authors:** Frederick I Burge, Beverley Lawson, Grace Johnston, Gordon Flowerdew

**Affiliations:** 1 Department of Family Medicine, Faculty of Medicine, Dalhousie University, Halifax, NS, Canada; 2 School of Health Services Administration, Dalhousie University, Halifax, NS, Canada; 3 Department of Community Health and Epidemiology, Faculty of Medicine, Dalhousie University, Halifax, NS, Canada

## Abstract

**Background:**

During the 1990s, health care restructuring in Nova Scotia resulted in downsized hospitals, reduced inpatient length of stay, capped physician incomes and restricted practice locations. Concurrently, the provincial homecare program was redeveloped and out-of-hospital cancer deaths increased from 20% (1992) to 30% (1998). These factors all pointed to a transfer of end-of-life inpatient hospital care to more community-based care. The purpose of this study was to describe the trends in the provision of Family Physician (FP) visits to advanced cancer patients in Nova Scotia (NS) during the years of health care restructuring.

**Methods:**

Design Secondary multivariate analysis of linked population-based datafiles including the Queen Elizabeth II Health Sciences Centre Oncology Patient Information System (NS Cancer Registry, Vital Statistics), the NS Hospital Admissions/Separations file and the Medical Services Insurance Physician Services database. Setting Nova Scotia, an eastern Canadian province (population: 950,000). Subjects: All patients who died of lung, colorectal, breast or prostate cancer between April 1992 and March 1998 (N = 7,212). Outcome Measures Inpatient and ambulatory FP visits, ambulatory visits by location (office, home, long-term care facility, emergency department), time of day (regular hours, after hours), total length of inpatient hospital stay and number of hospital admissions during the last six months of life.

**Results:**

In total, 139,641 visits were provided by family physicians: 15% of visits in the office, 10% in the home, 5% in the emergency department (ED), 5% in a long-term-care centre and 64% to hospital inpatients. There was no change in the rate of FP visits received for office, home and long-term care despite the fact that there were 13% fewer hospital admissions, and length of hospital stay declined by 21%. Age-sex adjusted estimates using negative binomial regression indicate a decline in hospital inpatient FP visits over time compared to 1992–93 levels (for 1997–98, adjusted RR = 0.88, 95%CI = 0.81–0.95) and an increase in FP ED visits (for 1997–98, adjusted RR = 1.18, 95%CI = 1.05–1.34).

**Conclusion:**

Despite hospital downsizing and fewer deaths occurring in hospitals, FP ambulatory visits (except for ED visits) did not rise correspondingly. Although such restructuring resulted in more people dying out of hospital, it does not appear FPs responded by providing more medical care to them in the community.

## Background

Canadians have become increasingly vocal about the need for improved care for the dying. In response, the Special Senate Committee on Euthanasia and Assisted Suicide[1] declared that Canadian governments should make end-of-life care or palliative care a top priority in the restructuring of the health care system. As the leading cause of adult deaths, it is estimated that 67,400 Canadians died of cancer in 2003[2]. The aging of the Canadian population will result in increasing numbers of individuals presenting to the health care system with advanced chronic illnesses such as cancer[3]. In one Canadian province, Nova Scotia, the number of cancer deaths is projected to increase by 27% from 2,259 in 1999 to 2,870 by 2010[4]. Although cancer is the leading cause of death in those who receive care from comprehensive palliative care programs, access to such programs is hugely variable[5,6].

As these calls for better care for the dying go out, hospital roles in Canadian communities have been redefined. Hospital restructuring has transferred many aspects of inpatient care to community-based care, including the end-of-life or palliative care of those with cancer. End-of-life care is defined broadly as all care provided to dying persons. Multiple providers are involved in such care and include generalist providers such as family physicians, community nurses and other primary care providers, hospital-based providers, specialists, specialist palliative care providers, volunteers and family. Unless otherwise stated "end-of-life care" reflects any or all of these kinds of care as received by dying patients. When warranted, we will refer to "palliative care program" (PCP) as the comprehensive, organized and specialized program of care for the dying. Such PCPs are often hospital based and include an inpatient palliative care unit, consultant nurses and physicians and may provide consultations and / or care in the community. End-of-life care has been provided in the community in the past, however, this health system restructuring has forced an even greater emphasis on this location for care.

Nova Scotia is a small, east coast Canadian province of just under one million people where the percentage of cancer deaths occurring outside of hospitals rose from 19.8% in 1992–93 to 30.2% in 1997–98, an increase of over 50%[7]. One direct result of this change, we feel, is the need for effective, available, continuous and increasingly complex care of the dying in the community. Options to provide this end-of-life care vary from the coordination and integration of existing community resources including family physicians to specialized palliative care program home support teams providing all the necessary visits. There are no free-standing, community-based hospices available to the dying in Nova Scotia.

While health system restructuring has increased community-based care, restructuring has also affected the context in which family physicians provide this primary medical care. In Nova Scotia, substantial health system restructuring occurred during the study years and initially capped physician incomes, restricted practice locations, downsized hospitals, provided new hospital-in-the-home capacity, initiated redevelopment of a provincial home care program and introduced drug co-payments for seniors receiving government drug benefits.

The purpose of this study was to describe the trends in the provision of family physician visits to those dying of the four cancers with the highest mortality in Nova Scotia and to whom, we believe, would be most frequently seen by a family physician, during the years concurrent with this health care restructuring. We hypothesized that, given our previous research showing the trends of advanced cancer patients spending more time out of hospital, and fewer dying in-hospital, family physician community-based services to them would increase.

## Methods

Study subjects included all Nova Scotians who died due to lung, colorectal, breast or prostate cancer from April 1, 1992 to March 31, 1998 as indicated on the Vital Statistics death certificate (International Classification of Diseases. 9^th ^revision [ICD9-CM]).

This population-based study involved the secondary data analysis of linked administrative health information. Individual level data were obtained from: (1) the Queen Elizabeth II Health Sciences Centre Oncology Patient Information System (OPIS) which encompasses the provincial Nova Scotia Cancer Registry (NSCR) and includes provincial Vital Statistics information, (2) the Nova Scotia Medical Services Insurance Physician Services (MSIPS), and (3) the provincial Hospital Admissions and Separates file (HAS). The MSIPS includes data pertaining to all visits provided by physicians in the province who are remunerated via fee-for-service schedules and for physicians who are paid alternatively that provide 'shadow' billing information. More than 96% of FPs in Nova Scotia were fee-for-service at the time of this study[8].

Health services were limited to the 'end-of-life (EOL)', defined in this study as the six months (180 days) prior to the date of death, or from the date of initial cancer diagnosis as recorded in the NSCR to death for persons living less than six months after diagnosis. Service fee codes (for fiscal years 1992–93 to 1995–96) and health service identification numbers (for fiscal years 1996–97 to 1997–98) were used to identify FP visits.

### Measures

All visits provided by a family physician were counted for each patient during their end-of-life including those in the FP office, patient's home, long-term-care facility (LTC), emergency department (ED), and hospital inpatient settings. Ambulatory visits were defined as all visits, including those to the emergency department, but excluding those made to a patient during a hospital inpatient stay or outpatient procedures. Two 'time of care' categories were created: regular hours (8:01 am to 5 pm), and after hours care (5:01 pm to 8 am, weekends, holidays). Because a common code was not available to identify the time of visit to hospitalized patients, all hospital inpatient visits were considered as occurring during regular hours.

In addition to the total length of inpatient hospital stay and the number of hospital admissions, we also examined the total number of specialty visits received by these patients. Demographic and clinical variables included sex, age, fiscal year of death, geographic region, time from diagnosis to death, and tumour site (lung, colorectal, breast or prostate).

### Analysis

Initial analyses focused on frequency counts and descriptive measures (central tendency, dispersion) of all FP visits, hospital admissions, length of inpatient stay and specialty visits, overall and by fiscal year of death. Each count was expressed as the number of visits provided per 100 "end-of-life (EOL) person-days". Linear temporal trends were assessed using negative binomial regression with a logarithmic link function linking the dependent variable (for example, FP visits, hospital admissions, inpatient stay) to fiscal year. Fiscal year was included as a linear predictor adjusting for age and sex, and with log (EOL person-days) as an offset variable. Assessment of a nonlinear trend was made by including fiscal year as both a linear predictor and a qualitative predictor. The Type 3 analysis of year as a qualitative predictor in this model relates to the nonlinear component of trend. Adjusted regression coefficients were exponentiated and reported as rate ratios (RR) with associated 95% confidence intervals (CI). All analyses were conducted using SAS software[9].

## Results

In total, 7212 Nova Scotians were identified as having died due to lung, colorectal, breast or prostate cancer over the six-year study period. Males comprised a larger proportion of deaths, as did adults aged 65 years and older, those who died due to lung cancer and survivors of at least 150 days from date of initial cancer diagnosis (Table 1). The number of deaths across fiscal years remained relatively stable.

**Table 1:** Characteristics of adults who died due to lung, colorectal, breast or prostate cancer in Nova Scotia between April 1, 1992 and March 31, 1998

**Characteristic**	**Number of deaths (%)**
**Fiscal year of death**	
1992/93	1142 (15.8)
1993/94	1162 (16.1)
1994/95	1243 (17.2)
1995/96	1245 (17.3)
1996/97	1241 (17.2)
1997/98	1179 (16.4)
**Sex**	
Female	3126 (43.3)
Male	4086 (56.7)
**Age group (years)**	
< 65	1740 (24.1)
65–74	2151 (29.8)
75–84	2247 (31.2)
85+	1074 (14.9)
**Cancer cause of death**	
Lung	3674 (50.9)
Colorectal	1223 (17.0)
Breast	1243 (17.2)
Prostate	1072 (14.9)
**Survival time (days)**	
<31	723 (10.0)
31–60	470 (6.5)
61–90	340 (4.7)
91–120	277 (3.8)
121–150	222 (3.1)
>150	5180 (71.8)
**Region of death**	
Halifax regional municipality	2293 (31.9)
Cape Breton Island	1153 (16.0)
All other regions of Nova Scotia	3751 (52.1)

In total, 139,641 visits or a median of 13 visits per patient (mean 19.4; standard deviation [SD] 20.3), were provided by FPs to patients during their end-of-life with 94% of patients receiving at least one FP visit. Variability across fiscal years was minimal, ranging from 92.9% receiving at least one FP visit in 1993–94 to 95.3% in 1997–98.

The majority of FP visits were provided to hospital inpatients (64%), followed by the office (15%), home (10%), the emergency department (5%) and long-term care (5%). Of visits provided to hospital inpatients, almost 71% were categorized as a 'subsequent hospital visit', which represents continuing in-hospital care. Other inpatient visits included initial hospital visits (4.7%), visits after four weeks (17.5%), supportive care visits (4.6%) and urgent or emergency care visits (2.5%).

**Figure 1:**
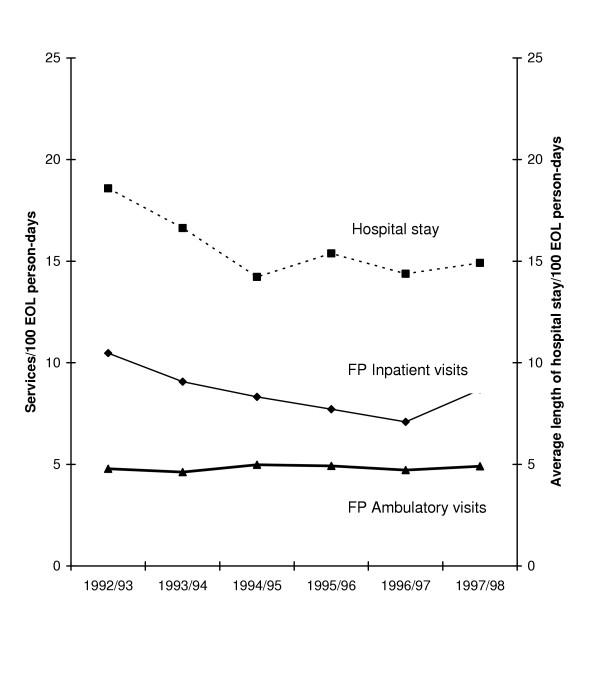
Family physician inpatient and ambulatory visits and length of hospital stay among advanced cancer patients over time

**Figure 2:**
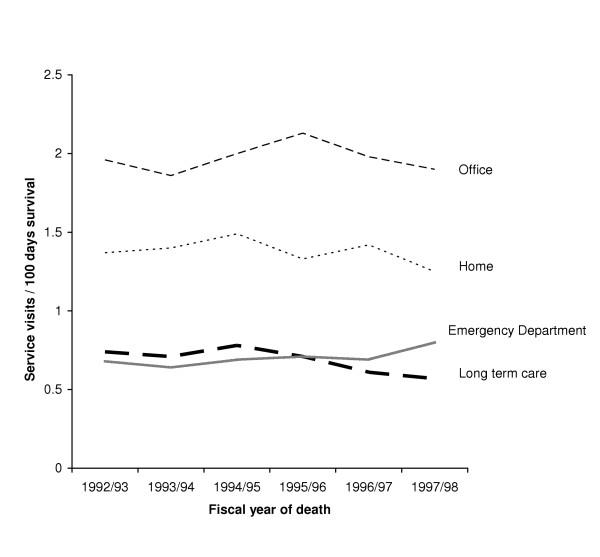
Ambulatory family physician visits to cancer patients by location over time

Temporal trends associated with ambulatory and inpatient FP visits per 100 EOL person-days are shown in Figure 1 along with the average number of days spent as hospital inpatient stay. Ambulatory visits by service location are illustrated in Figure 2. After accounting for age, sex and survival time, the total number of FP visits were found to have decreased significantly over the time period (p < 0.0001 declining from 15.3 visits per 100 EOL person-days in 1992–93 to 11.8 visits per 100 EOL person-days in 1996–97 followed by a small increase to 13.6 visits per 100 EOL person-days in 1997–98. This nonlinear trend is primarily due to the decline and then rise in the number of inpatient visits made by FPs over time. A closer examination of these inpatient visits by category (e.g., initial hospital visits, subsequent visits) did not reveal any major shift in the distribution of inpatient visit types over time. In contrast, total ambulatory visits remained relatively stable over the six-year time period with no evident significant time trends. Stratification of ambulatory visits by location of visit indicate a significant linear trend in emergency department visits over time (p < 0.01). After accounting for age and sex, patients in 1997–98 made 18% more emergency department visits than patients in 1992–93 (adjusted RR 1.18; 95% CI 1.05, 1.34). No association was evident across time for visits provided in the office, at home or within a long term care facility.

Examination of ambulatory FP visits provided during regular hours (8:01 am-5 pm) showed no significant change over time. However, ambulatory visits provided during 'after' hours (5:01 pm-8 am, weekends) were found to have increased significantly over the study period (p < 0.03). Compared to patients in 1992–93, patients in 1997–98 received 12% more ambulatory visits after hours (adjusted RR 1.12; 95% CI 1.01, 1.25). Although this increase represented a significant change over time, after hour visits were relatively few compared to the large number of other ambulatory visits and therefore exerted no impact on the overall temporal effect among all ambulatory visits.

Significant temporal trends were evident with respect to both the total number of hospital admissions experienced by the patient and the total number of days they spent as a hospital inpatient. A decline in hospital admissions was seen over time, from 1.2 admissions in 1992–93 to 1.1 admissions per 100 EOL person-days in 1997–98. Compared to 1992–93, patients in all subsequent years experienced fewer hospital admissions after accounting for sex and age. By 1997–98 patients experienced 13% fewer hospital admissions than patients in 1992–93 (adjusted RR 0.87; 95%CI 0.82, 0.93).

Over 85% of patients spent at least one day as a hospital inpatient. Patients spent on average a total of 22.7 days in hospital (SD 27.4; median 14 days; range 0–180 days) or 15.6 days per 100 EOL person-days. Total length of hospital inpatient stays declined from 18.6 days per 100 EOL person-days in 1992–93 to 14.8 in 1997–98. Results from the age and sex adjusted regression analysis indicate total length of hospital inpatient stays in 1997–98 were 21% shorter than experienced in 1992–93 (adjusted RR 0.79; 95%CI 0.71, 0.88).

In total, 82,575 visits were made to a medical specialty during the end of life. The number of visits ranged from one to 169, with a median of 11 visits per patient (mean 11.4; SD 13.5). Age and sex adjusted regression analysis indicate visits to a medical specialty did not change significantly over time.

## Discussion

Despite the move to a greater percentage of cancer patients dying out of hospital[7] and despite the findings of this study which show advanced cancer patients are spending more time out of hospital during the end-of-life, we have found no indication of increased family physician involvement in office, home or long-term care settings. There was an increase in FP visits in the emergency department. Many questions follow. By whom and how is the medical component of community-based end-of-life cancer care being provided? We have shown that the number of visits made to a specialist physician has not changed significantly over the same time period. Therefore, is care previously performed by family doctors now being offered by non-physicians? Might these patterns be influenced by changes in the provision of end-of-life care with an increased use of systemic therapies? Are patients receiving adequate and appropriate care at the end-of-life?

When we compare these trends to concurrent trends in the province of Nova Scotia for all types of patients, a number of interesting points emerge. For *all types *of patients in the province from 1992–1999, the total number of office-based, home and long-term-care visits has declined slightly[8]. This is not true for the patients in our study. FPs may be continuing to see cancer patients in the office despite reduced office visits for other types of patients in an attempt to ensure comprehensive care for those with this serious illness.

We expected to see an increase in home visits for those dying of cancer given the longer period people are spending out of hospital and the greater numbers dying at home. This was not the case. Home and long-term care visits among patients remained stable over time. This trend may have been facilitated by a revamped home care system in 1995 providing more nursing and assisted care visits in the home in the latter part of the decade perhaps reducing the need for family physician visiting.

Hospital visits declined quite substantially in the early years of our study and then increased in the final year. Provincial information suggests this trend was true for non-cancer patients as well[8]. Since both length of stay and the number of hospital admissions have declined, it is possible that those admitted in 1997/1998 were those who had been cared for longer in the community but who had reached a critical point where they were sicker than in the previous years and required more visits during these shorter stays. In other words, if patients in hospital had greater severity of illness, greater medical visit intensity may have been required. This may account for the rise in hospital visits in the last study year.

The increase in emergency department visits by family physicians parallels a rise during the study period among the general Nova Scotian population. (personal communication, M. Joyce, Department of Finance, Government of Nova Scotia). This greater emergency department utilization may be a reflection of reduced access to inpatient beds experienced by all patients, including those with advanced cancer.

The lack of increased visits made by family physicians in end-of-life care in the community is concerning when one considers the evidence that their participation is associated with a greater likelihood of home death [10-12] and less emergency department use[13]. It is not, however, surprising given the overall decline in comprehensiveness of care by individual family physicians in Canada[14].

It is important to note that the advantage of using provincial administrative health databases is that we have information for the entire population regarding cancer mortality and physician and hospital utilization. However, there is no clinical information on severity of disease, which would provide a much better understanding of factors influencing health service utilization. In addition, we do not have concurrent home care utilization data or private long-term care facility data to factor into our modeling.

## Conclusions

Despite health care restructuring of the 1990s which resulted in fewer days in-hospital for those dying with cancer in Nova Scotia, there was no concurrent increase in the family physician visits provided to the dying. This may represent a growing unmet need for community-based medical care. However, further research is needed to examine whether end-of-life-care needs are being met by other health providers such as specialized home palliative care nurses, or general home care nursing services.

## Competing interests

The author(s) declare that they have no competing interests.

## Authors' contributions

FB and BL participated in the conceptualisation and design of the project, the analysis and interpretation of the data, created the first draft of the article, and incorporated co-authors' comments into the final draft. GJ participated in the design of the project, the interpretation of data, and revising the manuscript. GF participated in the analysis and interpretation of data and the revising of drafts. All authors gave approval to the final version.

## Pre-publication history

The pre-publication history for this paper can be accessed here:


